# Understanding attitudes, barriers and challenges in a small island nation to disease and partner notification for HIV and other sexually transmitted infections: a qualitative study

**DOI:** 10.1186/s12889-015-1794-2

**Published:** 2015-05-02

**Authors:** O Peter Adams, Anne O Carter, Lynda Redwood-Campbell

**Affiliations:** Faculty of Medical Sciences, University of the West Indies, Cave Hill campus, St. Michael, Barbados; Department Community Health and Epidemiology, Faculty of Health Sciences, Queen’s University, Kingston, ON Canada; Department of Family Medicine, McMaster University, Hamilton, ON Canada

**Keywords:** Disease notification, Partner notification, Patient referral, Provider referral, Contract referral, HIV, Sexually Transmitted infection, Attitudes, Barbados

## Abstract

**Background:**

In Barbados sexually transmitted infections (STIs) including HIV are not notifiable diseases and there is not a formal partner notification (PN) programme. Objectives were to understand likely attitudes, barriers, and challenges to introducing mandatory disease notification (DN) and partner notification (PN) for HIV and other STIs in a small island state.

**Methods:**

Six key informants identified study participants. Interviews were conducted, recorded, transcribed and analysed for content using standard methods.

**Results:**

Participants (16 males, 13 females, median age 59 years) included physicians, nurses, and representatives from governmental, youth, HIV, men’s, women’s, church, and private sector organisations.

The median estimated acceptability by society of HIV/STI DN on a scale of 1 (unacceptable) to 5 (completely acceptable) was 3. Challenges included; maintaining confidentiality in a small island; public perception that confidentiality was poorly maintained; fear and stigma; testing might be deterred; reporting may not occur; enacting legislation would be difficult; and opposition by some opinion leaders.

For PN, contract referral was the most acceptable method and provider referral the least. Contract referral unlike provider referral was not “a total suspension of rights” while taking into account that “people need a little gentle pressure sometimes”. Extra counselling would be needed to elicit contacts or to get patients to notify partners. Shame, stigma and discrimination in a small society may make PN unacceptable and deter testing. With *patient referral* procrastination may occur, and partners may react violently and not come in for care. With p*rovider referral* patients may have concerns about confidentiality including neighbours becoming suspicious if a home visit is used as the contact method. Successful contact tracing required time and effort. With *contract referral* people may neither inform contacts nor say that they did not.

Strategies to overcome barriers to DN and PN included public education, enacting appropriate legislation to allow DN and PN, good patient counselling and maintaining confidentiality.

**Conclusions:**

There was both concern that mandatory DN and PN would deter testing and recognition of the benefits. Public and practitioner education and enabling legislation would be necessary, and the public needed to be convinced that confidentiality would be maintained.

**Electronic supplementary material:**

The online version of this article (doi:10.1186/s12889-015-1794-2) contains supplementary material, which is available to authorized users.

## Background

Disease notification (DN) is the reporting by a physician, other health care provider or laboratory of the occurrence of specified notifiable diseases to a designated public health agency [1]. Reporting is regulated by public health legislation. For sexually transmitted infections (STIs), partner notification (PN) is the process by which sex partners of infected persons are informed of their exposure to infection and offered testing, prevention counselling and treatment [2,3]. In the case of blood borne STIs, this would extend to needle sharing partners.

DN along with PN can ensure the proper follow up and management of individual cases by public health authorities and interrupt the chain of STI transmission [3,4]. On the population level DN by itself can be used for disease surveillance, allowing the monitoring of disease trends and thereby providing information necessary for the development of public health policy, resource allocation, and the assessment of the effectiveness of prevention and care programmes [4]. The Centers for Disease Control and Prevention (CDC) recommends that HIV/AIDS DN should be complete (recording ≥ 85% of cases), timely (≥66% of cases reported within 6 months) and accurate (≤5% duplicate case reports). A confidential name-based system is most likely to meet these criteria and fulfil the public health purposes for which the surveillance data are required [5]. However there has been concern that name-based HIV reporting might discourage people from getting tested because of fear that their names “might fall into the wrong hands” [6] and the CDC strongly promotes the concurrent availability of anonymous testing options [5]. Data from six states in the USA with a low HIV prevalence [7], New York State with a higher prevalence [8] and from Alberta, Canada another low prevalence area [9] suggest that the introduction of confidential name-based DN will not have a significant impact on testing rates. In the UK, voluntary HIV DN using a combination of soundex codes based on the surname and date of birth has proven efficient in identifying duplicates. The use of initials, date of birth, and gender together may also be efficient in excluding duplicates [10].

Notifiable disease reporting completeness is highly variable and is related to the disease, physician and patient concerns about confidentiality, physician knowledge of reporting procedures, administrative difficulties, and whether a passive or active surveillance system is in place [11-13].

Three main approaches to PN are recognized: patient referral, provider referral, and contract referral. *Patient referral* gives the index case the responsibility of contacting partners and asking them to present for treatment. *Provider referral* gives health care personnel the responsibility of obtaining the names and contact information of the partners of the index case and then contacting them. DN can facilitate this process. With c*ontract referral* the index case is encouraged to notify their partners, with the understanding that health service personnel will notify those partners who do not visit the health service by an agreed date [2,3]. PN identifies a high-prevalence population and should be part of any STI prevention and treatment programme [2,14,15]. A review of HIV partner counselling and referral services in high-income countries estimated that with provider referral 67% of named partners were notified, 63% of those notified were tested and 20% of tested individuals were newly diagnosed with HIV [2]. Provider referral or the choice between provider referral and patient referral, compared with patient referral for patients with any STI may increase the rate of partners presenting for medical evaluation [16,17]. However, a recent systematic review did not identify a single optimal strategy of PN for any particular STI as there were too few trials to allow consistent conclusions about the relative effects of provider, contract or other patient referral methods for different STIs [3]. For curable STIs both patients and doctors may prefer patient referral [18,19]. Provider referral is more costly than patient referral and may not be successful if it is perceived to threaten patient confidentiality [20]. The significant benefit of HIV treatment is a strong reason for encouraging individuals exposed to HIV to be tested [2]. Notified contacts may however suffer harms such as stress, emotional trauma, domestic violence, partnership dissolution, a loss of confidentiality, stigmatization and discrimination [2]. In a survey of HIV infected persons in Barbados 71% reported that they had self-disclosed their status to their steady partner, but only 26% to casual partners. Reasons given for nondisclosure included fear of stigma, discrimination, loss of confidentiality and rejection [21].

Barbados is an island of only 430 Km^2^. It has a population of 277,821 [22]. The estimated prevalence of Chlamydia is 11.3% and gonorrhoea 1.8% in people 18 to 35 years of age [23]. For HIV the estimated prevalence among 15 to 49 year olds is 1.2%. Between 1984 when the first HIV case was diagnosed and 2011 there have been 3559 people diagnosed with HIV, 2375 AIDS cases and 1643 deaths [24]. Voluntary counselling and confidential HIV testing using a code rather than name to identify specimens sent to the laboratory is available free through publicly funded polyclinics, and for a fee through private practitioners. Anonymous testing is not done [25]. Point-of-care HIV testing is not usually done, and collected blood is tested at a single public laboratory. Other STI laboratory testing is done at both public and private laboratories with specimens identified with the patient’s name. Since 2002 free antiretroviral treatment has been available to all HIV infected people through a centralised location [26]. However, STIs including HIV are not notifiable diseases in Barbados and there is no formal PN programme. There is limited published information on the attitudes and challenges to DN and PN in Barbados and other small island states, and these may be different from what is seen in larger countries. By understanding the likely attitudes of people in Barbados to DN and PN, public health policy can be developed that is culturally appropriate and more likely to be accepted. The objectives of this study were to interview a diverse group of people in Barbados in order to understand the likely attitudes, barriers, and challenges to introducing mandatory DN and PN for HIV and other STIs in a small island state.

## Methods

This was a focused ethnographic study conducted in 2005. This type of research not only describes behaviour but also tries to understand why people do what they do. A survey or quantitative approach would not generate the rich information that qualitative interviews could. The reporting of this study adheres to the RATS guidelines for qualitative studies [27].

### Sampling

Study participants from diverse backgrounds were interviewed. They ranged from health care workers directly involved in STI/HIV/AIDS work in Barbados to non-health care workers not directly involved in such work (Table 1). An attempt was made to ensure that study participants represented important sub-populations. Participants had to be resident in Barbados and be ≥16 years of age. An equal distribution between the two genders was a priority. Based on the saturation model, the aim was to conduct interviews until no new data emerged.

**Table 1:** **Description of study participants by category and gender**

**Category**	**Description**	**Number gender**
Health care workers (HCW)	5 physicians, 2 nurses involved in Public Health HIV care	4 male
		3 female
Government position (Govt)	1 physician, 1 social worker, other 4 persons affiliated to various non-medical organisations/commissions	2 male
		4 female
People living with HIV/AIDS (PLWHA)	3 people from organisations concerned with HIV, 1 person with HIV	3 male
		1 female
Nongovernmental organisations (NGO)	Representatives of youth organizations, 2 men’s groups, 2 women’s groups, 2 churches, 2 private sector business organisations and 2 other non-governmental organizations	9 male
		3 female

Study participants were selected by interviewing six key informants. Key informants were individuals identified as having good community contacts and knowledge. They were chosen through snowball sample technique on the recommendation of knowledgeable community members. The sampling method in both the key informant and the individual interviews were purposive. Individuals were selected based on their level of cultural knowledge of the topic.

### Data collection

A fieldworker was hired and trained to do the recruitment and interviews. The study participants were asked to read an information sheet, sign a consent form and fill out a personal demographic information sheet. Consent included permission to have the interviews tape-recorded and transcribed. Participant interviews were conducted individually using an open-ended semi-structured interview guide (Additional file 1). Transcriptions were labelled with a code.

Understanding of DN was first determined, and if necessary an explanation of DN was given and understanding checked again. The participant’s attitude towards DN for STI including HIV, opinion on the acceptability to society of DN for these conditions, rating of the acceptability to society on a scale of 1 (unacceptable) to 5 (completely acceptable), identification of groups that would support and oppose DN, and barriers and challenges to the implementation of DN were obtained. After this a review of the statements made was done to validate the researcher’s interpretation of data collected, and finally further comments were invited. Participant understanding of PN was then assessed, terms were defined and understanding re-assessed if needed. The same areas as for DN were explored for provider, patient and contract referral in turn. Participants then ranked the acceptability of the 3 methods of PN with the help of show cards, then a review of the statements made. Interviews took approximately 60 to 90 minutes each.

### Analysis and interpretation of the data

The 3 authors conducted independent analysis of transcribed participant interviews. Participants were categorised into demographic groups – health care workers (HCW), government position (Govt), people living with HIV/AIDS (PLWHA), and non-governmental organisations (NGO) which included representatives of youth groups, men’s groups, women’s groups, the private sector, and the church (Table 1). All authors analysed 3 interviews separately, identifying themes from the transcripts and developing their own coding framework. The codes were then compared and integrated into a common framework. All 29 transcripts were then coded using the common framework, and then amalgamated by category of participant and type of PN. Information from each category of participant and type of PN was analysed separately, then compared together so that triangulation of data occurred, and then a summary done. Quotes were chosen in order to illustrate a range of views from different participants. Medians were calculated for the estimated acceptability DN and the different forms of PN.

### Ethical approval

Ethical approval was received from the Institutional Review Board of the School of Clinical Medicine and Research (now the Faculty of Medical Sciences), Cave Hill Campus, University of West Indies.

## Results

Twenty-nine participants (16 males, 13 females) (Table 1), with a median age of 59 years (range 30 to 68) were interviewed.

### Current DN and PN practices

Few (HCW, Govt, PLWHA) were aware of current DN and PN practices. HIV/STI DN was described as an informal process rather than a legal requirement (HCW). HIV testing was done at a single public laboratory. Laboratory workers would not know the name of the patient as specimens were labelled with a code. When a positive test was obtained, the laboratory director contacted the doctor who requested the test, to obtain information on the patient (HCW, Govt). For HIV “*the present system of DN is actually quite good*” (HCW), while another person felt that it was not practical (PLWHA). For other STIs public sector polyclinics but not private physicians reported the number of cases diagnosed each week to the Ministry of Health (HCW).

Health care workers said that there was no legislation specifically governing PN, and current practices incorporated elements of patient, provider and contract referral. Patients were encouraged to reveal their status to partners and encourage them to come in for testing. One person said that if this failed they would ask for the partner’s name and inform the partner without revealing the name of the infected person. Another person said that a doctor or the public health nurse contacted partners and told them what infection they are exposed to.

#### Acceptability

##### Society’s acceptance of DN

The estimated acceptability by society of HIV/STI DN had a median score of 3 on a scale of 1 (unacceptable) to 5 (completely acceptable).

Making STIs notifiable diseases would be controversial as it dealt with a very personal and sensitive issue in a society that does not openly discuss such matters and tends to stigmatise persons with STIs (Govt). The difference between success and failure might depend on way it was *“marketed”* and the extent of an anticipated public “*outcry”* that would occur with its introduction (HCW). Some felt that DN might not be accepted because of a lack of trust and concerns about confidentiality. Acceptability would improve once it became clear that confidentiality would be respected (Govt). Others felt that society would accept HIV DN since there had not been a negative reaction to the current informal system, and people realised that HIV was an important problem (HCW, Govt).

Some (PLWHA) estimated that 50 to 75% would support DN but they would want it for everyone else except themselves. *“I think that there are people who would support it because they are of the perception that it will never apply to them, you know that”* (NGO).

##### Study participants’ acceptance of DN

Most participants were in support of HIV DN. HIV is a public health concern and DN would allow for appropriate policy to be developed to help manage it (Govt, NGO). The collection of names and addresses was necessary for accurate data as persons had the tendency of being retested when first diagnosed (Govt). It needed to be in place if the spread of HIV was to be slowed (NGO). Comments included that it was *“an important tool”, “long overdue*” (HCW), *“Barbados really needs it right now”, “I think it would work” (NGO), “a good idea because you have an opportunity to better track the disease” and “the church would certainly stand behind this”* (NGO). Although people had “*rights*” there are always exceptions such as the public interest (Govt).

A few were not in support of DN for HIV. One person said that it was already known that HIV was an epidemic, and could not see how DN would help anything (Govt) and another said that information between a patient and a doctor should remain confidential (PLWHA).

### Acceptability of PN

For HIV most felt that contract referral would be the most acceptable method of PN and provider referral the least acceptable method to society. When acceptability of each method of PN was rated on a scale of 1 (unacceptable) to 5 (completely acceptable) the median score was 4 for both contract and patient referral, and 3 for provider referral. Contract referral allowed shared responsibility for notification with the patient being given the first chance to inform the partner. Unlike provider referral it was not “*a total suspension of rights”* while taking into account that *“people need a little gentle pressure sometimes*” (HCW).

The acceptability of provider referral would be slightly better with STIs other than HIV. *“People don’t like to hear the news (about STI) but they don’t pull people out of the house”* (HCW).

#### Barriers and challenges to disease and partner notification

##### Confidentiality

For both DN and PN many noted that maintaining confidentiality would be especially challenging in a small island like Barbados where many people know each other. “*What I am wondering is how much confidentiality will be there considering that Barbados is so small and everybody know everybody*”. “*It is so hard to keep things private in Barbados (NGO*). In addition Barbadians were said to have an excessive interest in the business of others while being very secretive about their own health, resulting in a particularly difficult environment for implementing DN. “*Sociologically Barbadians are still very concerned about divulging even basic diseases like diabetes. Bajans (Barbadians) are very secretive, very conservative”* (NGO).

With DN and PN even an isolated breach of confidentiality might cause a significant loss of trust in the system. It may be sufficient to turn the system “*upside down*” (NGO). It would be difficult to identify who was responsible. In some cases it may be a spouse or friend. Even if not at fault, health care workers would take most of the blame.

There was some scepticism that the systems in place could maintain confidentiality. With DN access to the information might not be adequately restricted. People are afraid that the information “*will be put in a file in a government department where somebody could be the officer this year, then next year it is somebody else, or next month somebody goes on vacation and someone comes in and sees confidential information. People are afraid of that”* (NGO). Many were of the view that confidentiality was not well maintained in Barbados and the public therefore lacked trust in the system. Not all professionals respected confidentiality. “*People in private sector (banks) breach confidentiality, so why is this different?” “A lot of people will not believe that it is confidential. Nobody in Barbados trusts anyone else”* (NGO). *“I don’t think that officialdom is aware of what people feel. They just feel that there is no confidentiality in the system because they have had bad experiences.” “Because of our small society they are afraid that other persons would know, afraid that confidentiality would be breached”* (NGO). With PN a partner once informed may become angry and tell someone.

Judgmental attitudes of health care workers and a lack of trust in them may also be barriers.

“*Apart from being a very gossipy society we’re also a very judgemental society especially when it comes to morals and ethics and as you know sex and sexuality ...... which we don’t talk a lot about.... we judge people by their sexual practices a lot”* (Govt).

Stigma and discrimination made concerns about confidentiality even more important. “..*some people who are run out of their work places not because their employers say I can’t employ you but people are treated differently. .... I mean a man only must begin to lose weight and the first thing people (are going to) ask him ‘you got AIDS’, ‘you see so and so, he losing very much weight yuh!.’* (NGO).

##### Effect on HIV testing

People will not want to be tested because of concerns about confidentiality, and stigma, discrimination and embarrassment associated with infection (HCW, NGO).

*“It (DN) may appear to have short-term advantages… but people are not going to then come forward for any voluntary testing”. “There are some who would want to fly out of Barbados to get medical attention”* (NGO).

Mandatory PN may deter people from returning for their results. Some may resort to home HIV tests. People may delay accessing care and present late with “full blown” AIDS.

##### Historical concerns

Historical approaches to STI care provide negative examples. Confidentiality was lacking as there were identifiable STI clinics, and health care workers doing PN were easily identified.

##### Opposition

Politicians, prominent persons with a reputation to protect and other opinion leaders might oppose DN and PN. Despite the view that health care professionals would support legislation mandating DN one person felt that those working in STI clinics *“know that a blanket notification really inhibits people rather than helps”* (HCW). Persons engaged in high risk activities, and people living with HIV might also oppose mandatory DN. The church may be divided on the issue.

##### Legislation

There may be a lack of political will to enact legislation to enable DN. Health care workers would have to convince the politicians that that this is important. Some may feel it infringes human rights and there may be an orchestrated campaign against it via call in radio programmes.

##### Reporting may not occur

Doctors may not report the diseases. DN has not worked in the case of other diseases as doctors did not bother to report cases (HCW). People may feel that if they went to private practitioners the process may not apply (NGO).

### Partner notification barriers and challenges

Extra counselling would be needed to get patients to notify partners, or to give the provider permission to do so. Pre-test counselling was however often inadequate as doctors did not always have the skill or the time, and were often reluctant to refer patients to other professionals. The patient may not want the partner notified because of denial, fear and shame. There could be concern over partner reaction e.g. domestic violence (physical and mental), and a loss of economic support including being put out of the house.

The patient may not know the name or contact details of partners *as “one night stands”* were common and one interviewee believed that less than 50% of partners would be reached (NGO). This would also be true for sex workers and people who had sex while travelling.

#### Patient referral

With patient referral notification may not occur as some people would simply not care, some may not accept the responsibility, procrastination would occur and people may not notify partners despite promising to do so. It took courage to notify a partner, and many may not have this courage. “…*I have a difficulty in seeing that type of courage*” (Govt). As a result persons might rationalise the situation. “…*like if you going to have it she already got it so why worry*”, and many persons keep quiet and only reveal it in their dying days, or when they are so ill that it is noticeable (NGO). Giving bad news especially if it is face-to-face and revealing infidelity with multiple people to a close partner would be difficult. Partner reaction especially violence would be a concern.*“… if you have people with a short fuse, short tempers the person might grab the other person and want to lash out at the other person”. “I’ve heard stories of people being attacked for less”* (NGO).

With patient referral less than half of notified partners might come in for care (Govt). With provider referral they will know that a mechanism is in place for treatment and might be more inclined to get treatment (NGO).

### Provider referral

Although rated the least acceptable method to the public some might prefer provider referral. Not everyone would want to contact his or her partners themselves and it would reduce the possibility of violence and conflict associated with patient referral. However several challenges were identified.

Time and effort was needed for successful contact tracing, and without appropriate legislation difficulties could arise unless the patient gave permission. People would see it as a *breach of trust*. It is not *“a simple thing of saying all right you have been diagnosed that’s your wife let me go and tell her”* (HCW). Contacts may not welcome unknown health care workers knowing their sexual history and how to contact them.

Maintaining confidentiality would be a greater challenge compared to patient referral as contact tracing requires additional persons having access to the results. *“There are a lot of people involved, a great risk of confidentiality lost, and you have no guarantee at the end of the day, that the person is going to give you the correct list anyway”* (HCW). Given the small size of Barbados, people would be concerned that someone in the public health department with access to the information would know them (Govt). It would only be possible to keep the infected person’s identity secret if the contact had multiple partners. Additionally the contact tracer might reveal the name of the infected person, or might be pressured into doing so by a contact demanding to know who put them at risk. There may be hostility towards the contact tracer, especially if the name of the index case is not revealed. Family and neighbours may become suspicious if a health care provider associated with provider referral visits a home. Notification had to be done discretely without neighbours being able to guess what is happening, but people would be concerned that over time in a small place like Barbados the word would get out. With tuberculosis, a notifiable disease, people did not like it when the public health nurse visited the home, so the same may be true for STI, particularly HIV.

The patient may not reveal the names of partners because of concerns about confidentiality, stigmatisation, judgmental attitudes of health care workers and violence; or they may simply not know the names. One or two persons may be left off the list of contacts, especially if there is something threatening about those partners. There would be no way to force a person to disclose the name of their partners. Alternatively an angry patient might give names of persons who were not contacts. Even if the name was obtained the contact may not come in to be tested.

#### Contract referral

With contract referral a health care worker doing the notification may be seen as breaking confidentiality. A patient may have “*valid reasons*” for not notifying a partner and it may be seen as “*prying into people’s personal affairs”* (NGO). One person felt that it was best to tell patients that either patient or provider would inform the contact.

Determining the correct length of time to allow the patient to notify partners could be a challenge. Some persons will feel that they were not given enough time, some may need extra time as partners may be difficult to locate, but too much time could result in a delay in the partners being informed, and during that period they could become infected. People had a *tendency not to honour verbal contracts*, and could always deny that a contract was made. Many would neither inform their partners nor get back to the health care worker to say that they did not.

#### Strategies to make disease and partner notification work

Trust has to be created and fear reduced before DN or provider referral legislation is introduced. It was felt that a carefully planned public education campaign letting people know there were plans to introduce DN and PN, stressing the importance and reassuring the public that confidentiality would be maintained and attitudes would be non-judgmental was necessary. The required *public education* would take a long time. “*They would have to be absolutely certain that it would go as written, that is nobody would be snickering or pointing fingers at them*” (NGO). People would accept it only if confident that confidentiality was assured (NGO, Govt). For the information campaign to be effective the public had to be ‘*bombarded with information’* over a prolonged period (HCW). *“A little jingle on the radio wouldn’t do it”* (NGO). Public education messages however do not reach all people (PLWHA), and specific groups would have to be targeted (NGO). In addition to using the mass media, education should be taken to the schools and workplace (NGO). Education should be done at the community level, with a focus on young people and the lower income groups (NGO).

*“It would have to be clearly explained, let people understand that these numbers are necessary and only the smallest possible circle of persons will have this information. In other words it will not be a case where any clerical officer or any person in any government department will be able to pull out a file and see a person’s information”* (NGO).

##### Maintaining confidentiality

In order to maintain confidentiality education of staff was also needed. There had to be repeated training of staff dealing with the information and just not a one off effort (Govt). The consequences of breaching confidentiality such as the public losing trust in the system had to be made clear (NGO). Penalties would be needed to sanction those who breach confidentiality. With provider referral one had to be careful that the provider visiting a home is not known by the community to be associated with STI contact tracing. Alternative methods would have to be considered. *“I would imagine that people would have to be contacted by telephone, more so because you can’t have people traipsing up to people’s houses in vehicles that people identify to be government vehicles or persons”* (NGO).

##### Legislation

Legislation sanctioning DN and provider referral would have to be enacted.

## Discussion

Study participants were generally in favour of mandatory DN however they felt that the public would be less accepting. They felt that both contract and patient referral would be acceptable forms of PN in Barbados, but had concerns about the effectiveness of patient referral. Stigma and discrimination were significant concerns from a societal perspective, and for any method to be acceptable maintaining confidentiality would be important.

### Effect of mandatory DN on HIV testing

While the benefits of HIV DN and PN were appreciated by study participants there was fear that less people would come forward for testing or test results or may not provide the names of contacts largely due to concerns about confidentiality and the stigma associated with infection. Research indicates that people may provide more names for provider referral if they believe confidentiality is assured [20]. However, several studies from other countries suggest that DN regulations are a minor factor in HIV testing decisions with testing rates remaining the same after mandatory name-based DN was introduced [7-9], even if prior to the implementation there was a fear that rates would decrease [9]. Fear of receiving a positive test result and the assumption of being uninfected remain major reasons for not being tested [6]. However the situation could differ in Barbados because its small size makes confidentiality challenging to maintain, people are secretive about their health, and there is the perception confidentiality has been breached in the past. Confidentiality is particularly important in small societies with conditions that generate much stigma and fear. If confidentiality is breached or if patients believe that it may be breached they may not come forward for testing and both the individual and the public may suffer [28]. As encouraging people to become aware of their HIV/STI status is a major public health objective the introduction of an anonymous testing option should be considered.

### Maintaining confidentiality

Participants suggested that there should be repeated training of staff in maintaining confidentiality, the consequences of even an isolated breach in confidentiality to public trust should be made clear and penalties should be introduced for those breaking confidentiality. In addition there was concern that unsecured DN information might be accessed by persons visiting the office where the records were kept. The CDC suggests that surveillance records should be located in a physically secured area and be password and computer encryption protected, and access should be restricted to a minimum number of surveillance staff. In addition when surveillance data is made available for epidemiologic analyses names should be removed [5]. Adoption of these measures should help in maintaining trust. With DN the acceptability of using soundex codes and date of birth rather than actual names were not specifically explored by this study. While acceptable in larger countries [10] there may be concerns in a small island.

With provider referral it was also felt that over time in a small island contact tracers visiting homes would become known to the community and neighbours observing them would become suspicious. In a UK survey respondents felt that a mobile telephone text message, private email and private letters requesting the person to attend a clinic, rather than a message informing them that they have a STI would be good methods of PN [18].

### Completeness of DN

DN will provide accurate data only if reporting is complete. Legislation regulating DN would ensure that health care workers are protected when they reveal the requested information, and would provide a mechanism for the information be forwarded on the physician’s initiative. It would allow for the results of point-of care tests, which are likely to become more prevalent in the future, to be captured. Similarly legislation would be needed to facilitate provider and contract referral. Maintenance of trust requires patients to be informed of the reporting system before being tested.

In the USA reasons cited for health-care providers and laboratories not reporting notifiable diseases include a lack of knowledge of which diseases are reportable, the legal requirement to report these diseases and how to report; an assumption that someone else will report the case; intentional failure to report to protect patient privacy; and insufficient reward for reporting or penalty for not reporting. Interventions aimed at reducing these barriers have had limited success [29]. It is essential that an active HIV surveillance system in Barbados be maintained with the physician being contacted when a positive test is obtained by the laboratory. Inactivity by health departments can be a contributing factor to under-reporting [11].

### Partner notification acceptability and effectiveness

Contract referral and provider referral were chosen as the most and least acceptable methods respectively to the public. Contract referral was seen as preserving patient autonomy to some extent, while still allowing the provider to act if the patient did not thus increasing effectiveness. Other surveys have found that for STIs people may indicate that patient referral would be preferred over provider referral if they were to test positive [18], but the method preferred may vary by type of partner (close or casual) [20]. In Barbados, infected people are more likely to report that they had informed a regular (versus a casual partner) of their status, and some continued to have unprotected sex without doing so [21].

Patient referral may be less effective than provider or contract referral [16,17]. In a randomised controlled trial in North Carolina, USA 50% of partners of HIV infected people were notified in the provider referral arm but only 7% by patient referral despite a law requiring partners to be notified [16]. In our study, participants suggested several reasons why infected people may have difficulty with patient referral. In addition to concerns about violence, loss of economic support, shame in having to admit unfaithfulness to a regular partner and a loss of confidentiality, not being able to locate partners easily, a lack of concern, not liking to give bad news and procrastination were mentioned. Patients should be trained to instruct sex partners sensitively about their potential risk of infection [30] and this may be helpful in overcoming some of these barriers. Dual referral where both the patient and provider jointly notify the partner of exposure [31] may be another strategy to use. However some emotional distress and anger are to be expected if a partner believes that he or she has been unknowingly exposed to HIV [2].

### Limitations

A survey of the public, including people receiving a STI diagnosis would be needed in addition to this study to more completely explore attitudes to DN and PN.

## Conclusions

There was both a concern that mandatory DN and PN would adversely affect testing and a recognition of the benefits of their introduction. The introduction of DN and PN would require appropriate legislation and would have to be preceded by a sustained education and awareness campaign explaining the importance of DN and PN and how the system will maintain confidentiality. The latter would include measures to physically secure surveillance data, repeated staff training, penalties for breaching confidentiality and in the case of provider referral a discrete contact method. Extra resources would be needed for counselling and the contact tracing required with provider referral.

## Additional file

Additional file 1:
**Interview guide.**

## Abbreviations

CDC: Centers for disease control and prevention
DN: Disease notification
Govt: Government position employee
HCW: Health care worker
NGO: Nongovernmental organization representative
PLWHA: Person working with People Living with HIV/AIDS
PN: Partner notification
STI: Sexually transmitted infection
