# Supplementary material for: Understanding attitudes, barriers and challenges in a small island nation to disease and partner notification for HIV and other sexually transmitted infections: a qualitative study
Source: BMC Public Health. 2015 May 2;15:455. doi: 10.1186/s12889-015-1794-2 (PMC4450455; doi:10.1186/s12889-015-1794-2)
Supplement: Additional file 1: — Interview guide. [file 12889_2015_1794_MOESM1_ESM.doc]

**INTERVIEW GUIDE**

**ATTITUDES TO HIV DISEASE NOTIFICATION AND DISEASE REPORTING IN BARBADOS**

DATE:

TIME:

NAME:

CODE:

LOCATION:

Hello, my name is Monique Springer and I would like to thank you for your time today to meet with me. I am a research assistant affiliated with the School of Clinical Medicine and Research, UWI, and I will be spending about 30 minutes with you today to ask you some questions. They are questions that the research team hope will help us to understand what people in Barbados think about methods to control sexually transmitted infections (STI)/HIV/AIDS. These questions are not personal questions. We do not expect you to discuss your own private issues. We only want you to express your opinions about the situation in Barbados in general.

Firstly, we will review the consent form before signing it. The key points about the consent are:

- You can withdraw from the interview at any time
- You can decide that you do not want to answer any question
- We will be tape recording this interview for accuracy. I will also be taking notes to help me keep track of where we are as we talk. Later, we will make a typed version of our talk. At that time, your name and anyone else’s name you mention will be removed and the tape will be destroyed. This will ensure that nothing you say can be identified back to you.
- Any information that is presented will be presented without a name attached to it and presented as a group. (like woman vs men’s opinions etc.)

Do you have any questions about the consent?

Collect signed consent form.

Now I want you to complete this information sheet. This will help us to describe generally (not individually) the types of people that we interviewed.

Before we start, have you ever been interviewed before as part of a research project? (Get information about previous interviews and what the process was like. Describe how the process may be different or similar to past experiences)

This type of project is done by face-to-face interviewing. It allows us to get information on a subject from different types of people like yourself who may know a lot about how things run in your community.

My role today is to try to get you to think about and discuss some issues related to certain aspects of STI/HIV/AIDS in Barbados. Do you know what I mean by STIs?

HIV/AIDS is a problem seen all over the world. Barbados is no exception. It is estimated that 2% of people in Barbados have HIV. We know that there are important ways to control infections like STI/HIV/AIDS. One is called **disease notification** (DN) and the other is called **partner notification** (PN). Many countries around the world use these methods to reduce the spread of STI/HIV/AIDS. At this time, Barbados does not have these methods formally in place.

1. Have you ever heard of disease notification? (If yes, what is your understanding of this process? What do you think is involved in doing this?)

____________________________________________________________________________________________________________________________________________________________________________________________________________________________________________________________________________________________________________________________________________________________________________________________________________________________________

If they answer exactly correctly, go to questions.

Let me tell you a little bit about **disease notification** first. This is currently done in Barbados for many infectious diseases but not for sexually transmitted diseases or HIV/AIDS. Let’s say that someone has tested positive for HIV (meaning a person has HIV). With disease notification, the health care worker would be required by law to report this to a central authority. Sometimes the results from the lab could go automatically to a central agency. This information is confidential but reporting does involve a breach of confidentiality between physician and patient. The only other people who would see the details of the case would be ones directly involved in controlling the spread of infection. For example, people who are counting the number of cases need to know names/addresses so that they don’t double count the information from the lab and the treating doctor. However, even those people who are analyzing and reporting the numbers about the infection would *not* be given names/addresses. Knowing the correct numbers and trying to understand the numbers would help to create public health programs and see how well these programs are working. It could be very important step to keeping people healthy in Barbados. For example, seeing that many new cases are occurring in young women would lead to the design of programs to help young women prevent themselves from getting infected. Once such programs are in place, seeing the rate of new infections in young women fall would indicate that the new programs are working well.

Before we continue, it would be helpful for me to make sure that you have understood the concept of disease notification so that I can accurately obtain your opinions.

2. Would you mind summarizing what disease notification is in your own words? Do you have any other questions about disease notification? (answer any questions as they occur)

______________________________________________________________________________________________________________________________________________________________________________________________________________________________________________________________________________________________________________________________________________________________

3. Now that we have discussed disease notification for STI/HIV/AIDS, what is your reaction to it?

______________________________________________________________________________________________________________________________________________________________________________________________________________________________________________________________________________________________________________________________________________________________

4. From a societal perspective, how acceptable do you think disease notification of STI/HIV/AIDS would be in Barbados? What do you think of it? (Follow up on barriers and challenges as they come up in interview, asking for examples of what might be more acceptable instead)

______________________________________________________________________________________________________________________________________________________________________________________________________________________________________________________________________________________________________________________________________________________________

5. I would like to ask you to rate societal acceptability on a scale of 1 to 5. (1=never acceptable under any circumstances to 5=completely acceptable). Write down number chosen- if scale seems to be very different than earlier comments, could probe in more detail as to why.

**Number** _________

**Explanation** ______________________________________________________________________________________________________________________________________________________________________________________________________________________________________________________________________________________________________________________________________________________________

Many countries have programs in place to try to control infections like STI/HIV/AIDS. There are some people in favor of new programs and others who are not so comfortable with change.

6. Which groups in Barbados do you think are the ones most likely to support something like disease notification for STI/HIV/AIDS? (If unable to answer, give probes like church groups, politicians, business people, tourism industry etc. Probe for further information, like how to gain their active support)

______________________________________________________________________________________________________________________________________________________________________________________________________________________________________________________________________________________________________________________________________________________________

7. Which groups may be most against it? (Probe for how this group may be otherwise convinced)

______________________________________________________________________________________________________________________________________________________________________________________________________________________________________________________________________________________________________________________________________________________________

(REVIEW STATEMENTS MADE ABOUT DISEASE REPORTING WITH PARTICIPANT AS A SUMMARY HERE)

8. Are there any other comments?

____________________________________________________________________________________________________________________________________________

________________________________________________________________________________________________________________________________________________________________________________________________________________________________________________________________________________________

Thank you. Let’s talk about the idea of **PARTNER NOTIFICATION** now.

9. Have you heard of Partner Notification? (if yes, what is your understanding of this process? What do you think is involved in doing this?)

______________________________________________________________________________________________________________________________________________________________________________________________________________________________________________________________________________________________________________________________________________________________

If answer exactly correct, including all 3 ways, go to question.

**Partner notification** means that if a person has a positive HIV/STI test, their recent sexual partners are contacted. They are told that they also may be infected or may be in danger of becoming infected. There are different ways of doing this. I would like your thoughts on some of them. I am going to describe 3 different possible ways of notifying partners. I will ask you to rate the acceptability on a scale of 1 to 5. (1 =never acceptable under any circumstances to 5 =completely acceptable) Please think in terms of what would be acceptable for the majority of people in Barbados.

The first way is called *provider referral*.

The second is called *patient referral*.

The third is called *contract referral*.

**Provider referral** means that someone from the health care system notifies partners. There are a few steps involved here so I will review it step by step. If a person has a positive test for HIV or another sexually transmitted disease, for example, then the health care provider (usually the doctor) would have to notify the authorities, like the public health department of the Ministry of Health. The public health authorities would then contact the person who tested positive and ask them for a list of the recent sexual contacts. The public health department would then get in touch with the list of contacts and tell them that an anonymous recent sexual contact has an infection and they too need to go to their doctor to get care. It is important that partners get care because this helps both the individual person get good treatment and also helps to control the spread of disease. Also, getting treated for other sexually transmitted diseases can decrease the chance of getting other STD’s/HIV.

I have given you a lot of steps here and sometimes repeating the steps helps to clarify the definition.

10. Could you please give me the steps involved in provider referral and I will review any questions that you have?

______________________________________________________________________________________________________________________________________________________________________________________________________________________________________________________________________________________________________________________________________________________________

Great, thanks.

11. Now, thinking about people in Barbados again, from a societal perspective, how acceptable do you think this would be for HIV/AIDS? What do you think? (follow up on barriers, challenges)

______________________________________________________________________________________________________________________________________________________________________________________________________________________________________________________________________________________________________________________________________________________________

12. Could you please rate, from a societal perspective, this idea of provider referral (for HIV/AIDS) on a scale? On a scale of 1 to 5, how acceptable do you think this would be? (1=never acceptable under any circumstances to 5=completely acceptable). If previous comments and the rank on the scale are very different, probe again here.)

**Number** ____________

**Reason**

______________________________________________________________________________________________________________________________________________________________________________________________________________________________________________________________________________________________________________________________________________________________

13. For those people who would not be comfortable with this, what do you think their main reasons would be?

______________________________________________________________________________________________________________________________________________________________________________________________________________________________________________________________________________________________________________________________________________________________

14. Would attitudes to this be different for other sexually transmitted infections? Would it change your acceptability score?

______________________________________________________________________________________________________________________________________________________________________________________________________________________________________________________________________________________________________________________________________________________________

15. Why do you think some people might be against this idea? Who or what groups might be against this? Probe here again if unsure, examples of groups

______________________________________________________________________

________________________________________________________________________________________________________________________________________________________________________________________________________________________________________________________________________________________

The second type of referral is called **patient referral**. This means that the health care provider (doctor/nurse) encourages the infected person to notify their partners on his/her own to come to the health care system for care. In this case, no disease reporting has to occur although reporting for other reasons may occur. (like counting and doing statistics).

16. Do you need to clarify this definition?

______________________________________________________________________

17. Now, thinking about people in Barbados again, from a societal perspective, how acceptable do you think this would be for HIV/AIDS? What do you think? (follow up on barriers, challenges)

______________________________________________________________________________________________________________________________________________________________________________________________________________________________________________________________________________________________________________________________________________________________

18. On a scale of 1 to 5, how acceptable, from a societal perspective, do you think this would be for HIV/AIDS? (1 =never acceptable under any circumstances to 5 =completely acceptable)

______________________________________________________________________________________________________________________________________________________________________________________________________________________________________________________________________________________________________________________________________________________________

19. For those people who would not be comfortable with this, what do you think their main reasons would be?

______________________________________________________________________________________________________________________________________________________________________________________________________________________________________________________________________________________________________________________________________________________________

20. Would people actually notify their partners? Would the partners come in for care?

______________________________________________________________________________________________________________________________________________________________________________________________________________________________________________________________________________________________________________________________________________________________

21. Would societal attitudes to this be different for other sexually transmitted infections?

______________________________________________________________________________________________________________________________________________________________________________________________________________________________________________________________________________________________________________________________________________________________

22. Who (what groups) might be against this? (probe here again if unsure, examples of groups). Why do you think some people might be against this idea?

______________________________________________________________________________________________________________________________________________________________________________________________________________________________________________________________________________________________________________________________________________________________

The last type of partner notification is called **contract referral.** This is almost a mix of the other two**.** This means that the health care provider (doctor/nurse) gives the infected person the choice of notifying their partners on his/her own. If the infected person chooses not to or if the contact does not go to get care by a certain date, however, then the health care system will notify the contacts directly.

23. Do you need to clarify this definition?

______________________________________________________________________________________________________________________________________________________________________________________________________________________________________________________________________________________________________________________________________________________________

24. Now, thinking about people in Barbados again, from a societal perspective, how acceptable do you think this would be for HIV/AIDS? What do you think? (follow up on barriers, challenges)

______________________________________________________________________________________________________________________________________________________________________________________________________________________________________________________________________________________________________________________________________________________________

25. On a scale of 1 to 5, from a societal perspective, how acceptable do you think this would be for HIV/AIDS? (1 =never acceptable under any circumstances to 5 =completely acceptable)

______________________________________________________________________________________________________________________________________________________________________________________________________________________________________________________________________________________________________________________________________________________________

26. For those people who would not be comfortable with this, what do you think their main reasons would be?

______________________________________________________________________________________________________________________________________________________________________________________________________________________________________________________________________________________________________________________________________________________________

27. Would attitudes to this be different for other sexually transmitted infections?

______________________________________________________________________________________________________________________________________________________________________________________________________________________________________________________________________________________________________________________________________________________________

28. Who (what groups) might be against this? (probe here again if unsure, examples of groups) Why do you think some people might be against this idea?

______________________________________________________________________________________________________________________________________________________________________________________________________________________________________________________________________________________________________________________________________________________________

29. After hearing about 3 different possibilities for partner notification, I would like to know which one do you think is most acceptable? (I have 3 cards in my hands. Each one represents the 3 different possibilities that we have just discussed. I will ask you to take the card that you think would be most acceptable in Barbados. Take the card which is least acceptable in Barbados.)

______________________________________________________________________________________________________________________________________________________________________________________________________________________________________________________________________________________________________________________________________________________________

(REVIEW STATEMENTS MADE ABOUT PARTNER NOTIFICATION WITH PARTICIPANT AS A SUMMARY HERE)

30. Are there any other comments that you would like to make?

______________________________________________________________________________________________________________________________________________________________________________________________________________________________________________________________________________________________________________________________________________________________

31. I would like you to open your mind and brainstorm for a few minutes. Do you have any ideas about other ways of tracking STI/HIV/AIDS and getting partners in for care which may be even more acceptable? Perhaps something that we haven’t even discussed today. Maybe you have an idea that would work even better than what we have described?

______________________________________________________________________________________________________________________________________________________________________________________________________________________________________________________________________________________________________________________________________________________________

32. For those who would be aware of current legislation, can you suggest any changes in legislation that would contribute to controlling STI/HIV/AIDS?

______________________________________________________________________________________________________________________________________________________________________________________________________________________________________________________________________________________________________________________________________________________________

33. Are you aware of any documents or resources that would provide information on this topic? If yes, do you know where I could obtain a copy?

______________________________________________________________________________________________________________________________________________________________________________________________________________________________________________________________________________________________________________________________________________________________

34. There any other groups of people whom you feel we should interview?

______________________________________________________________________________________________________________________________________________________________________________________________________________________________________________________________________________________________________________________________________________________________

35. We only have a few more minutes until we will finish the interview. I wonder if you could summarize in a few sentences your last thoughts about disease reporting and partner notification in Barbados

______________________________________________________________________________________________________________________________________________________________________________________________________________________________________________________________________________________________________________________________________________________________

36. Is there anything that you would like to tell us about the interview to make it better for the next person who we interview? Should anything in the interview be changed?

______________________________________________________________________________________________________________________________________________________________________________________________________________________________________________________________________________________________________________________________________________________________

I would like to thank you very much for your time today. Hopefully, your ideas can help towards controlling a very important disease in Barbados.
